# Alcohol, Inflammation, and Microbiota in Alcoholic Liver Disease

**DOI:** 10.3390/ijms24043735

**Published:** 2023-02-13

**Authors:** Marija Dukić, Tijana Radonjić, Igor Jovanović, Marija Zdravković, Zoran Todorović, Nemanja Kraišnik, Bojana Aranđelović, Olga Mandić, Višeslav Popadić, Novica Nikolić, Slobodan Klašnja, Andrea Manojlović, Anica Divac, Jasna Gačić, Milica Brajković, Svetlana Oprić, Maja Popović, Marija Branković

**Affiliations:** 1 University Hospital Medical Center Bežanijska Kosa, 11000 Belgrade, Serbia; 2 Faculty of Medicine, University of Belgrade, 11000 Belgrade, Serbia

**Keywords:** alcohol, inflammation, microbiota, alcoholic hepatitis

## Abstract

Alcoholic liver disease (ALD) is a consequence of excessive alcohol use. According to many studies, alcohol represents a significant socioeconomic and health risk factor in today’s population. According to data from the World Health Organization, there are about 75 million people who have alcohol disorders, and it is well known that its use leads to serious health problems. ALD is a multimodality spectrum that includes alcoholic fatty liver disease (AFL) and alcoholic steatohepatitis (ASH), consequently leading to liver fibrosis and cirrhosis. In addition, the rapid progression of alcoholic liver disease can lead to alcoholic hepatitis (AH). Alcohol metabolism produces toxic metabolites that lead to tissue and organ damage through an inflammatory cascade that includes numerous cytokines, chemokines, and reactive oxygen species (ROS). In the process of inflammation, mediators are cells of the immune system, but also resident cells of the liver, such as hepatocytes, hepatic stellate cells, and Kupffer cells. These cells are activated by exogenous and endogenous antigens, which are called pathogen and damage-associated molecular patterns (PAMPs, DAMPs). Both are recognized by Toll-like receptors (TLRs), which activation triggers the inflammatory pathways. It has been proven that intestinal dysbiosis and disturbed integrity of the intestinal barrier perform a role in the promotion of inflammatory liver damage. These phenomena are also found in chronic excessive use of alcohol. The intestinal microbiota has an important role in maintaining the homeostasis of the organism, and its role in the treatment of ALD has been widely investigated. Prebiotics, probiotics, postbiotics, and symbiotics represent therapeutic interventions that can have a significant effect on the prevention and treatment of ALD.

## 1. Introduction

Alcoholic liver disease (ALD) is caused by chronic excessive alcohol consumption [1]. Alcohol has been used since ancient times, and many tests and studies have confirmed its hepatotoxic effect [1,2]. Its chronic use implies intake of more than 40 g per day for men or more than 20 g per day for women [3,4]. More than 40 g of alcohol is precisely defined as more than 375 mL of 13 vol % wine or more than 1 L of 5 vol % beer [4]. Some studies have shown that even taking smaller doses, between 12–24 g per day, increases the risk of liver cirrhosis compared to those who do not consume alcohol [5]. At the world level, alcohol is the most significant risk factor for disability and death [6]. The risk of death depends on various factors, and one of them may be age, as is the case with alcoholic hepatitis [3]. The share of the consequences of alcohol consumption as a cause of death is about 4.0% for women and about 7.6% for men worldwide [7]. What is interesting is that alcohol is not used to the same extent in developed and underdeveloped countries. Additionally, there is a difference in relation to geographical localization. Thus, research has shown that alcohol use per capita has increased by about 10% in Asia and Africa in the last 25 years, while alcohol consumption for this period has decreased by 1% in Europe and America [8].

ALD occurs in about 20% of alcoholics, with a higher frequency in women [1]. Many studies have shown that women develop ALD when consuming smaller amounts of alcohol compared to men, and that they suffer from more severe forms of the disease. It is known that by consuming the same amount of alcohol, women achieve a higher alcohol blood level [9,10,11]. This is because women have a smaller constitution and a lower percentage of water in the body, which results in a smaller volume for alcohol distribution [9,10]. First-pass metabolism of alcohol has been shown to be reduced in women who consume it due to differences in alcohol dehydrogenase activity. In addition, estrogen affects liver function through receptors on liver cells. In an experiment on rats, it was shown that alcohol increases the expression of these receptors in male rats, which results in the proliferation of hepatocytes. In contrast, in female rats, the expression of these receptors under the influence of alcohol is not increased and apoptosis of hepatocytes is dominant in relation to proliferation [12]. Additionally, in the same experimental animals, studies have shown that estrogen stimulates the continuous release of growth hormone in female rats which then increases the activity of alcohol dehydrogenase, favoring the accumulation of acetaldehyde and the occurrence of ALD [13].

Obese and patients with chronic liver diseases, such as hemochromatosis, nonalcoholic steatohepatitis (NASH), and chronic hepatitis C and B virus infection, have a higher risk of developing the alcoholic liver disease [14,15,16,17,18]. Genetic factors also determine the risk of developing this condition [14].

**Figure 1:**
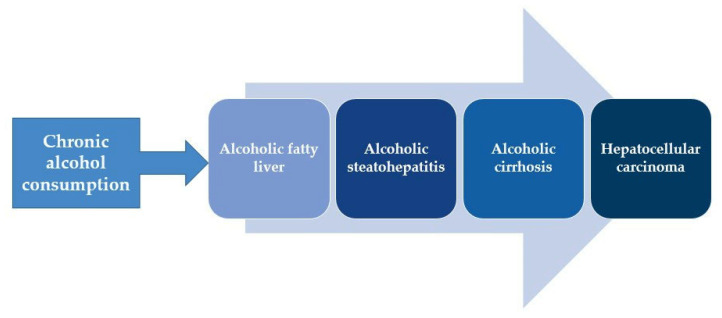
Modalities of alcoholic liver disease.

ALD is a term that encompasses several modalities: alcoholic fatty liver disease (AFL), and alcoholic steatohepatitis (ASH), which leads to liver fibrosis and cirrhosis [1,4] (Figure 1). Cirrhosis of the liver is the last stage of liver damage that is irreversible and occurs in about 20% of cases. The risk of occurrence of cirrhosis depends on the amount and duration of alcohol abuse, gender, age, the presence of other chronic diseases, nutrition, environmental factors, and genetic factors [19]. After several years, hepatocellular carcinoma (HCC) can also occur as a complication. It is estimated that HCC develops in 2–3% of the cases [20,21,22].

**Figure 2:**
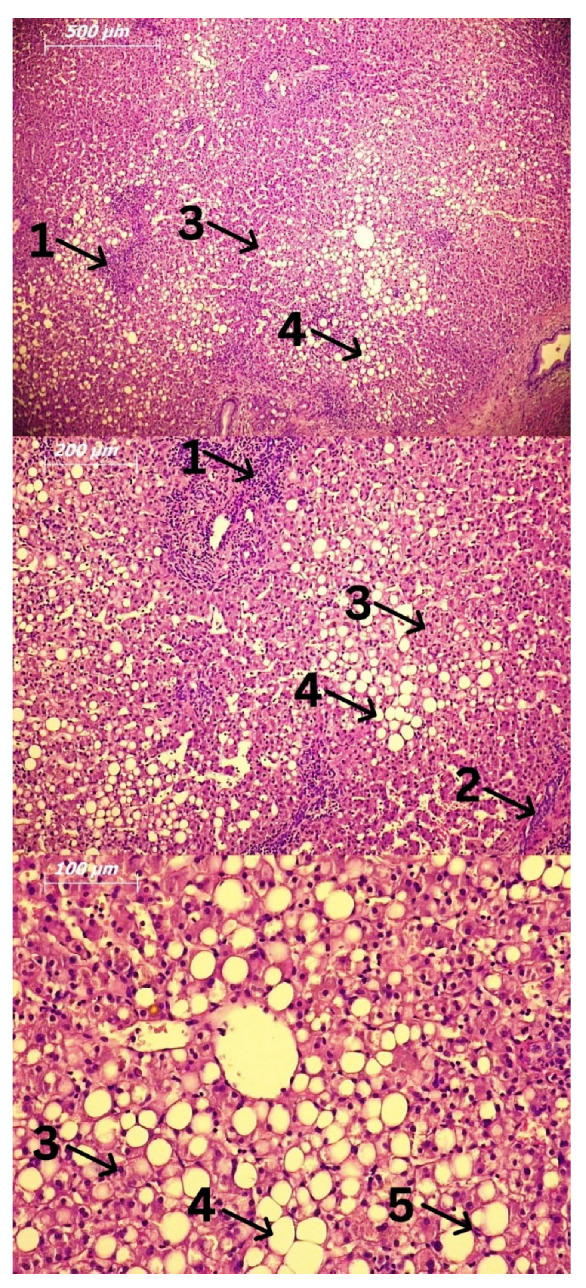
Fatty liver biopsy. Hematoxylin and Eosin (H&E) staining. Legend: 1: Lymphocytes; 2: Focal necrosis; 3: Ballooned hepatocytes; 4: Fat accumulation; 5: Kupffer cells.

AFL is characterized by steatosis, primarily due to the accumulation of triglycerides in hepatocytes (Figure 2). They can be accumulated in the form of micro- and macro-vesicles and disrupt the architecture of the cell [4]. If AFL progresses, this process is related to the development of inflammation, consequent damage of hepatocytes, and a phenomenon called ballooning [23]. This progression occurs in 20–40% of people who chronically consume alcohol [19]. ASH usually progresses slowly over time and is characterized by the development of fibrosis in response to chronic inflammation. If the progression takes place quickly, alcoholic hepatitis (AH) may develop with or without liver failure [23]. Additionally, AH can occur as a result of worsening of other stages of alcoholic liver disease, not only steatohepatitis. Patients who develop AH have the fastest progression of fibrosis, and in them, apoptosis and necrosis of hepatocytes are more pronounced compared to ASH [23].

## 2. Alcohol Metabolism

**Figure 3:**
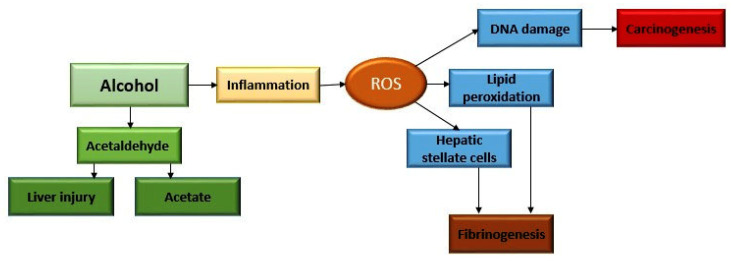
Metabolism and mechanism of harmful effects of alcohol.

Alcohol metabolism takes place mostly in the liver [24]. It begins under the influence of alcohol dehydrogenase, and in the presence of oxygen and coenzyme NADPH. In this way, a highly reactive and toxic metabolite called acetaldehyde is formed [24]. The reaction of acetaldehyde synthesis takes place in the cytosol of hepatocytes, and it is further metabolized under the influence of acetaldehyde dehydrogenase in the mitochondria to acetate. The well-known cytochrome P450 2E1, or CYP2E1, which is located in the endoplasmic reticulum and mitochondria, participates in alcohol metabolism [24,25]. Its activation by chronic alcohol use leads to the formation of reactive oxygen species (ROS). This is how the so-called alcohol-induced inflammation occurs. Evidence for this is that inhibition of CYP2E1 by chlormethiazole improves ALD and reduces the carcinogenic effect of acetaldehyde in experimental animals [26,27]. In addition, the so-called microsomal ethanol-oxidizing system (MEOS) was defined and discovered in the middle of the last century by C. S. Lieber and L. M. DeCarli [28]. Chronic excessive use of alcohol leads to the acceleration of alcohol metabolism via MEOS, precipitating the formation of toxic metabolites and faster tissue and organ damage [28]. Acetaldehyde is toxic and carcinogenic and leads to structural and functional damage to cell organelles [29]. For example, when mitochondrial damage occurs, adenosine triphosphate (ATP) production through the respiratory chain decreases, and ROS are generated [29] (Figure 3).

They further lead to changes in the structure of protein molecules, lipid peroxidation, and damage to DNA molecules [30,31]. In addition, they can lead to post-translational modifications, such as methylation, phosphorylation, and acetylation. Accumulation of ROS subsequently change macromolecules causing the occurrence and progression of already existing liver damage [31] (Figure 3).

## 3. Cells Involved in the Pathogenesis of Alcoholic Liver Disease (ALD) and Alcoholic Steatohepatitis (ASH)

### 3.1. Kupffer and Circulating Monocytes

**Figure 4:**
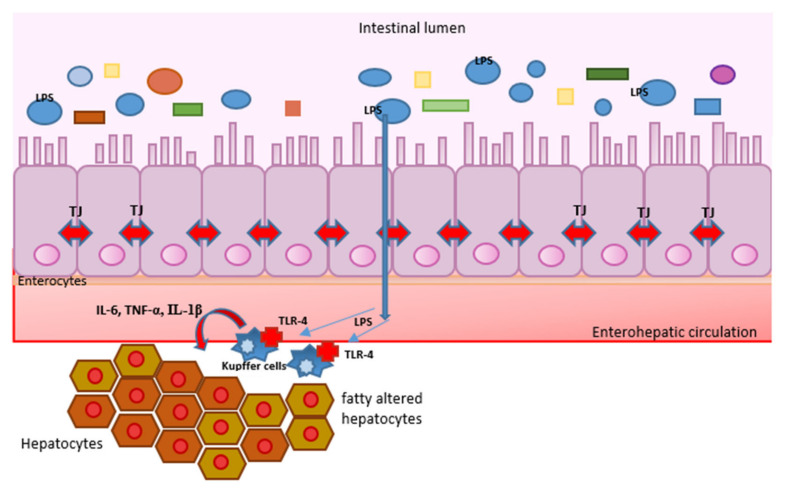
Cells which are involved in pathogenesis of alcoholic liver disease. In the intestinal lumen is the intestinal microbiota, the source of molecules which human organism recognizes as pathogen-associated molecular patterns (PAMPs). The role of lipopolysaccharide (LPS) of Gram-negative bacteria is best studied. It passes through damaged tight junctions (TJ) to the enterohepatic circulation, then to the liver, where it is recognized, among others, by Kupffer cells using Toll-like receptors class 4 (TLRs). In response to this stimulation, Kupffer cells secrete proinflammatory cytokines (IL-6, TNF-α, IL-1β) that lead to hepatocyte damage and the onset or progression of alcoholic liver disease.

In ASH, there is an increase in the number of hepatic macrophages, both resident Kupffer cells and circulating monocytes infiltrating the liver tissue [32]. After chronic ethanol use, Kupffer cells are sensitized and activated by molecules that will be discussed later, which trigger the production of cytokines and chemokines and initiates an inflammatory response [33]. These two types of cells are also called M1 macrophages. They produce abundant cytokines, such as IL-1β, IL-18, TNF, IL-12, IL-23, etc. There are also M2 macrophages, but their number increases in allergies and in the process of tissue repair after damage [34] (Figure 4).

### 3.2. Neutrophils

Drinking stimulates liver infiltration by these cells, which enhances the inflammatory response, promotes hepatocyte damage, and may be responsible for the progression of ASH to AH [35]. This accumulation of neutrophils is stimulated by cytokines and chemokines, while the degree of infiltration correlates with the severity of the disease. Interestingly, some studies have shown that neutrophils in this process can perform a protective role by promoting the repair of damaged tissue [36]. Neutrophils examined in AH have impaired phagocytic and bactericidal activity, and the potential therapeutic role of granulocyte colony-stimulating factor (G-CSF) was investigated [37,38].

### 3.3. T Lymphocytes

One role of T lymphocytes is in the presentation of antigens to cells of the immune system. This role belongs primarily to the CD4+ subpopulation of T lymphocytes. The TH1 subclass produces IFN-γ, IL-2, and TNF, which lead to the activation of macrophages and potentiates liver cell damage. The TH17 subclass promotes liver damage and inflammation via IL-17 and tissue recovery via IL-22. Cytotoxic T lymphocytes, or CD8+ T lymphocytes, lead to the death of hepatocytes that are damaged by a direct cytotoxic effect, but also by the activation of Kupffer cells and macrophages [39,40,41].

### 3.4. Hepatocytes and Hepatic Stellate Cells

Hepatocytes, as the basic cells of the liver parenchyma, are damaged by the previously mentioned molecules and cells in the inflammatory process, and in response, they release chemokines and cytokines that promote neutrophil infiltration and the development and progression of ALD [35]. It has been proven that they synthesize macrophage migration inhibitory factor (MIF), a pluripotent chemokine that contributes to the progression of ALD [42,43]. The main factors of this process are CXCL1 and IL-8 [44]. Their concentration is elevated in alcoholic hepatitis and determines disease severity [44]. Higher levels of CXCL1 have been shown in a mouse model of steatohepatitis, especially in those who received a higher amount of alcohol during the study, and who had a high-fat diet [34,35].

IL-8, also called CXCL8, is a pro-inflammatory cytokine that performs the role of neutrophil chemotaxis factor [45]. It performs this role by binding to chemokine receptors (CXCR1 and CXC2) [46]. In this way, neutrophils lead to inflammation in other tissues and organs, and in the liver. When this happens in people who have ALD, the condition can progress to ASH and AH [47]. Several studies have been conducted on the mouse model, in which it has been proven that by blocking CXCL1 and CXCL2, the development of alcoholic hepatitis and the occurrence of neutrophil infiltration are absent. Peptducin proved to be a substance that has this role. It also reduces the transcription of CXCL8 and other pro-inflammatory cytokines, such as CXCL1 and TNF [48].

Hepatic stellate cells are carriers of the process of damage reparation in the liver, i.e., of the fibrosis process [49]. They are activated in response to the chronic inflammation that exists in ASH due to chronic alcohol use. Neighboring Kupffer cells are responsible for their activation, but they can also be activated directly by alcohol and its toxic metabolites. In response to inflammation, they produce an extracellular matrix together with portal fibroblasts, thereby leading to fibrosis [49]. NK cells perform a role in the revers process by leading to apoptosis of hepatic stellate cells, but it is interesting that alcohol can also directly block their influence and promote the process of fibrosis [50,51].

## 4. Pathogen-Associated Molecular Patterns (PAMPs), Damage-Associated Molecular Patterns (DAMPs), Toll-like Receptors (TLRs), and Inflammation

Drinking triggers inflammation through two types of molecules called pathogen-associated molecular patterns (PAMPs) and damage-associated molecular patterns (DAMPs) [52,53]. Both are recognized by pattern-recognition receptors (PRRs), and thus, the inflammatory cascade begins. The most famous of this group of receptors are Toll-like receptors (TLRs), of which there are several classes. They can recognize both exogenous and endogenous pathogens and are expressed by both immune and liver parenchymal cells [54].

PAMPs are products of microorganisms that reach the liver via the lymphatic system and portal circulation. In the case of Gram-negative bacteria, it is lipopolysaccharide (LPS), which has been proven to be one of the best stimulators of TLRs [55]. In the case of chronic excessive use of alcohol, the permeability of the intestinal barrier increases, and the translocation of intestinal microbiota products takes place more easily into the lymph flow and portal circulation [56]. In this way, PAMPs reach the liver, where they activate Kupffer cells, which then activate infiltrating monocytes, stellate, and other cells. As a result of these activities, cytokines and chemokines that characterize the inflammatory response are produced. PAMPs most often through TLR4 cause NF-κβ activation and the release of CC-chemokine ligand 2 (CCL2) and IL-8, which then cause infiltration of the liver by macrophages and neutrophils [56]. In addition, TNF and IL-6 are released, which also have a pro-inflammatory effect. Their increased concentration is measured in acute AH and is associated with a worse outcome [56]. On the other hand, in the absence of infection, so-called sterile inflammation occurs, and it is characterized by integrity damage and death of cells [57]. On this occasion, DAMPs are released, most commonly ATP, adenosine, DNA, uric acid, heparin sulfate fragments, and others. Alcohol by its direct toxic effect promotes cell death in various ways, predominantly by activating the mitochondrial apoptotic program and damaging the endoplasmic reticulum [58]. This phenomenon is most pronounced in AH [59].

After the activation of TLRs, the post-translational processing of the pro-forms of cytokines and the release of their active forms occurs, e.g., IL-8 which has already been discussed and IL-1β. IL-1β acts through the TLR4 receptor by activating NF-κβ as previously reported [56]. Its active form is released only after the activation of a complex called the inflammasome [60]. Inflammasome is a multiprotein complex that belongs to the innate immune response and activates caspase-1, the effect of which, among other things, creates the active form of IL-1β from the inactive one [60,61]. This cytokine has a pro-inflammatory effect, autocrinely increasing its concentration and stimulating the release of TNF. This process increases the sensitivity of hepatocytes to apoptotic factors [62,63]. Research has shown that by blocking the receptor for IL-1β, the regeneration of hepatocytes is stimulated [60]. Additionally, IL-1β promotes liver fibrosis by activating hepatic stellate cells via matrix metallopeptidase 9 (MMP9) [64]. This form of the inflammasome, which involves the activation of caspase-1, is called the canonical inflammasome. Non-canonical inflammasome includes the CASP4/11-GSDMD complex, which activation causes cell death through lysis [65,66]. It is very important in infection-induced cell death with the consequent release of DAMPs and the initiation of an inflammatory response. This reaction can be so intense that it triggers systemic inflammatory response syndrome (SIRS), which can be seen in the rapid progression of ASH to AH. This often happens in infections caused by Gram-negative bacteria when LPS activates the CASP4/11-GSDMD pathway, the activity of proteolytic enzymes is triggered inside hepatocytes, then the release of DAMPs and pro-inflammatory cytokines. If hepatic macrophages and stellate cells succumb to cell lysis, systemic dissemination of endotoxin occurs with the development of SIRS and sepsis [52,65,66].

## 5. What Is the Role of Intestinal Microbiota in the Mentioned Processes?

In the gastrointestinal tract, there are more than a trillion microorganisms, primarily bacteria, but also viruses, fungi, protozoa, and archaea. They participate in digestion, metabolism of various substances, and endogenous production of alcohol, but also in host immunity [67]. Endogenous production of alcohol by bacteria of intestinal microbiota implies a fermentation process that takes place independently of the exogenous introduction of alcohol. This has been proven, for example, in an obese mouse model [68]. The endogenous production of alcohol has been proven by measuring the level of alcohol in the blood of people who have not consumed it in a certain period. Bacteria in anaerobic conditions switch to a mixed-acid fermentation pathway, and the main product of this pathway is alcohol [69]. Alcohol produced in this way is also metabolized by alcohol dehydrogenase, which is most active in the liver and gastrointestinal tract [70]. It is interesting that Zhu et al., showed that the level of endogenous alcohol in the blood is higher in obese patients with proven non-alcoholic steatohepatitis than in obese patients who do not have it. These results are explained by differences in the composition of the microbiota [70].

The connection between the intestine and the liver is bilateral: microorganisms and their products reach the liver through the lymph flow and portal blood flow, and on the other hand, the bile produced in the liver through the biliary tract is secreted into the intestine. Alcohol consumption causes changes in the composition of the intestinal microbiota, making it less diverse, reducing the number of bacteria that have a beneficial effect on health, and increasing the number of those that can have a harmful effect. This refers primarily to Gram-negative bacteria [71]. In addition, chronic excessive use of alcohol increases the possibility of overgrowth of Candida species [72]. The intestinal barrier is the first point of defense of the organism against the penetration of pathogenic microorganisms and their products into circulation [73]. It possesses components of the innate and acquired immune system, such as neutrophils, secretory immunoglobulins, and T lymphocytes. Its integrity is impaired in alcoholics [73]. According to some data, more than half of alcoholics have proven intestinal barrier dysfunction and intestinal dysbiosis [74]. This increases the possibility of translocation of products of microorganisms, but also of live bacteria, into the systemic circulation, which will reach the liver. This phenomenon increases the possibility of ALD [74]. Increased permeability of the intestinal barrier, or the so-called “leaking gut”, is caused by the effect of alcohol on the tight junctions that are connected to the intestinal epithelial cells [75].

The intestinal microbiota is very diverse, and its composition differs depending on the part of the digestive tract and the number of bacteria it contains. For example, the colon has the richest microbiota [76]. Two main families of bacteria make up the intestinal microbiota, namely *Firmicutes*, as a representative of Gram-positive bacteria, and *Bacteroidetes*, which belong to Gram-negative bacteria [77,78]. The share of these two families in the composition of the intestinal microbiota is individual and differs in many conditions [79]. Apart from the two mentioned, several smaller families contribute to the diversity of the intestinal microbiome. Less than 0.1% of the microbiome consists of potentially pathogenic bacteria, such as *Escherichia coli*, *Campylobacter jejuni*, and *Bacteroides fragilis*. It has been proven that intestinal dysbiosis can perform a role in the development of inflammatory bowel diseases (IBD), autoimmune and metabolic disorders, and so on [80,81].

In ALD, the existence of small intestinal bacterial overgrowth (SIBO) and a decrease in the number of bacteria from *Lactobacillus* species have been proven [82,83]. They produce bactericidal substances that maintain the homeostasis of the intestinal microbiota and prevent the reproduction of pathogenic bacteria, such as *Salmonella* and *Shigella* species. Short-chain fatty acids (SCFAs), which include butyrate, acetate, and propionate, are produced as products of their metabolism. The role of SCFAs is to maintain the integrity of the intestinal barrier, and, in addition, they are a source of energy for intestinal epithelial cells and have an immunological role [84,85,86].

As we said, the interaction between the intestine and the liver is bilateral. Bile acid is important in the pathogenesis of liver diseases, in which intestinal dysbiosis also performs a significant role. Bile acids perform a role in fat emulsion [87]. In addition, they are signaling molecules that bind to G protein-coupled receptors and regulate lipid and glucose metabolism. In hepatocytes, primary bile acids are conjugated, which allows them to be reabsorbed at the level of the terminal ileum. A smaller part is not a subject to this recirculation, and, in the distal parts of the intestine, are converted into secondary bile acids under the influence of bacteria through the processes of hydroxylation and esterification [87]. Additionally, bile acids affect the composition of intestinal microbiota. *Clostridium*, *Lactobacillus*, *Bifidobacterium*, and others have an enzyme that deconjugates primary bile acids and enables their excretion through the digestive tract. Bile acids can have a protective role, i.e., prevent intestinal barrier dysfunction and maintain gut eubiosis. Secondary bile acids are more toxic to intestinal and hepatic cells, and studies have shown that their concentration is elevated in the feces and serum of alcoholics [88]. Intestinal epithelial cells and hepatocytes express the farnesoid X receptor (FXR) that recognizes the mentioned acids. Upon binding and activation of the receptor, the endocrine hormone fibroblast growth factor (FGF 19) is released, which inhibits the de novo synthesis of bile acids. However, when there is intestinal dysbiosis, the level of FGF 19 decreases, and an increase in bile acid synthesis occurs, thus secondary toxic bile acids are produced in a higher percentage than primary ones [89].

The role of PAMPs in the development of ALD has already been discussed, and LPS has been singled out as the most significant. It is a component of the outer membrane of Gram-negative bacteria and is also called endotoxin. Its concentration is elevated in ALD in humans, but also in experimental animals. LPS activates inflammasomes in the liver and triggers a local immune response in the intestines.

In addition, PAMPs also include Gram-positive bacteria-related peptidoglycans and lipoteichoic acid, and lipopeptides. They similarly lead to the initiation of an inflammatory response [90].

## 6. Therapeutic Interventions in Alcoholic Liver Disease (ALD) Involving the Microbiota

Given the importance of gut microbiota homeostasis in the pathogenesis of ALD, therapeutic options targeting it seems promising. It has been proven that the use of prebiotics, probiotics, postbiotics, and symbiotics can lead to the improvement of chronic liver diseases, ALD, and liver cirrhosis among others [91].

Probiotics are a group of non-pathogenic microorganisms whose role is to modulate and maintain homeostasis of the intestinal microbiota. They can reduce inflammation in the case of alcohol-induced liver inflammation and prevent “leaky gut”. For example, this is demonstrated using #BCL3 which includes Bifidobacterium breve, B. infantis, B. longum, L. acidophilus, L. paracasei, L. bulgaricus, L. plantarum, and Streptococcus thermophilus [92,93,94]. Prebiotics are food ingredients that are not digestible and help intestinal peristalsis and stimulate the growth of certain bacteria. Some studies have shown that prebiotics repair alcohol-induced liver damage in a mouse model by reducing bacterial overgrowth. Postbiotics or microbe-derived metabolites can also be used in the treatment of ALD [93,95].

Fecal microbiota transplantation (FMT) involves the transplantation of part of the microbiota of a healthy person to a person suffering from certain diseases. For example, the role of FMT in patients with AH and liver cirrhosis was investigated and satisfactory results were obtained. In a study conducted by Bajaj et al., 20 patients with liver cirrhosis of the most common viral etiology (predominantly HCV) with or without data on chronic alcohol use were included. The patient received a single FMT enema with or without antibiotics. It was concluded that FMT is a safe and well-tolerated procedure, it increases the diversity of intestinal microbiota and the number of beneficial bacteria. Patients who had FMT had significantly lower chance of developing AH [96]. Another study followed the 1-year survival of patients with AH who had FMT, where as many as 87.5% of patients who had FMT survived one year, compared to 33.5% of patients who were controls [97].

A promising approach in the treatment of ALD is also an attempt to rehabilitate the previously disturbed homeostasis of bile acid metabolism, which was proven in the mouse model of ethanol-induced liver damage. Return of homeostasis using a non-tumorigenic variant of FGF19 improves intestinal barrier function and reduces liver damage. Obeticholic acid (OCA) is an FXR agonist and has been proven to prevent intestinal vascular barrier disorders, which is why it has a potential place in the treatment of nonalcoholic fatty liver disease (NAFLD), while there is still insufficient data for ALD [98,99].

## 7. Conclusions

In the modern world, chronic alcohol use is a growing problem, and ALD and its modalities require great attention. ALD can represent a condition that shows chronicity and progress slowly to terminal liver damage, that is liver cirrhosis, but it can also have a rapid progression to AH and potential liver failure. Alcohol metabolism produces toxic metabolites that cause a whole cascade that involves the activation of the body’s defense cells, then the release of pro-inflammatory molecules that cause damage to hepatocytes and promote the development and progression of ALD to ASH and AH. The stimulus for this can be a product of bacteria from the impaired intestinal microbiota, which is a process more common in alcoholics. Intestinal dysbiosis and “leaky gut” promote the occurrence of alcohol-induced liver inflammation, which is why it makes sense to use preparations that maintain microbiota homeostasis, such as prebiotics, probiotics, postbiotics, and symbiotics. Considering that most of the studies were conducted in animal models, the definitive conclusions are still limited. New research projects are necessary to enable a better understanding of these processes in humans. This will increase the possibility of adequate prevention of the occurrence and progression of ALD, and the occurrence of its complications.

## Data Availability

Not applicable.
